# Pedagogical Content Knowledge as a Lens for Transforming Teaching in Medical and Health Professions Education

**DOI:** 10.1007/s40670-025-02325-8

**Published:** 2025-03-20

**Authors:** Peter J. Boedeker, Nancy P. Moreno, Alana D. Newell, Atul Maheshwari, Matthew A. McMillin, Samantha P. Tippen, Malford T. Pillow, Andrew D. Bergemann

**Affiliations:** Baylor College of Medicine, Houston, USA

**Keywords:** Pedagogy, Active learning, Faculty development, Teaching, Pedagogical content knowledge

## Abstract

Pedagogical content knowledge (PCK) is the separate, topic-specific, specialized knowledge of educators that integrates subject matter expertise with effective teaching strategies in response to an identified learner misconception or to optimize teaching. Although PCK has been used for decades as a framework for understanding the often-tacit ability of an educator to educate well, little has been done to bring this framework into medical and health professions education. We provide examples of PCK in various contexts, including large -group lectures, problem-based learning sessions, and bedside teaching. Finally, we present a tool for capturing instances of PCK that can be used to facilitate an educator’s self-reflection and development.

## Introduction

Medical and health professions education currently lacks a well-defined conceptual framework for instructional decision-making. Although various publications outline characteristics of effective educators in this field [1, 2], these do not fully capture the process educators use when navigating learner uncertainties and misconceptions. Pedagogical content knowledge (PCK) offers such a framework. Developed primarily outside of medical and health professions education, PCK provides insights into the thought processes educators use to tailor their teaching to specific learners and contexts. Being explicit about PCK in medical and health professions education can deepen our understanding of effective teaching and support the development of exceptional educators.

In this paper, we explore the evolution of PCK, drawing primarily on literature from the fields of childhood and adolescent education. To translate PCK into the context of medical and health professions education, we demonstrate how educators’ PCK is expressed across diverse settings, including lecture halls, problem-based learning sessions, and bedside teaching. Additionally, we introduce a simple tool designed to capture instances of PCK, promoting reflection and facilitating knowledge sharing among educators.

## Illustrative Scenario

Imagine you are a medical or health professions student. During a lecture, the instructor pauses and presents the class with a brief case description and three possible treatment options. You and your classmates are prompted to respond independently with your selected treatment decision using polling software, selecting option A, B, or C. Responses come in and are projected for the class to see: 30% select A, 20% select B, 50% select C. Seeing these results, the instructor states that C is correct and then proceeds to the slide on the next topic.

Alternatively, imagine this same scenario but instead of immediately progressing to the next slide, the instructor reiterates the information from prior slides to explain why options A and B are incorrect and why C is the best answer.

Again, imagine the same scenario, but after the instructor sees that 50% of respondents are selecting incorrect responses, the instructor asks learners to pair up and discuss the case, having each pair of learners discuss the question. The instructor then calls upon three different pairs to describe the rationale behind their answer of A, B, or C, respectively, and provides clarifying information as needed.

In these three alternatives to the same scenario, there is one important difference: the behavior of the instructor. In the first scenario, the instructor simply recognizes that learner answers to the poll question were variable and provides a correct answer without considering whether the knowledge of the students or wording of the question should be re-examined. In the second and third scenarios, the instructor recognizes the variability of responses, identifies possible misunderstood or miscommunicated content, and then makes a pedagogical decision on how to close the knowledge gap. In the second scenario, we see the reiteration of content; in the third scenario, the instructor implements an active learning strategy (a variation of think-pair-share) with an additional opportunity to check for understanding [3].

The educator’s thought process and instructional response to a perceived misconception or disparity in knowledge depend jointly on two broad knowledge categories, pedagogical knowledge and content (subject matter) knowledge, and are influenced by the learning context. Pedagogical knowledge is the knowledge of and ability to use different teaching strategies to teach new concepts or skills and bridge gaps in learners’ understanding of, or abilities to apply, content. Content knowledge pertains to the subject being taught and includes both the details of the specific content for a given learning session and an understanding of how the content being taught in that moment fits into the broader subject area. Combining these two knowledge domains allows the instructor to identify the gap in knowledge (content knowledge) and consider how to address the learning gap (pedagogical knowledge). The learning context then acts as a moderating factor; whether a specific learning strategy is optimal will depend on the constraints and affordances of the learning context. Such constraints and affordances may include the time remaining in a session, the experiences of the learners, or the setting of learning, e.g., in a classroom versus in a clinic. When pedagogical knowledge and content knowledge are productively combined to enhance learning, the product is its own type of knowledge: PCK.

Recognizing PCK as a framework for understanding instructional decision-making is increasingly important as medical and health professions education moves toward active learning. Active learning in medical education has been shown to produce improved learning outcomes and is preferred by learners when compared to passive lecturing [4]. Using active learning, misconceptions or knowledge gaps are readily identifiable because of increased use of formative assessment and dialogue between learners and instructors. Therefore, in active learning contexts, the ability of an educator to flexibly respond to identified misconceptions or knowledge gaps, i.e., exhibit PCK, is critical. However, much of the rich discussion and research on PCK has existed in primary (grades K–5), secondary (grades 6–12), and post-secondary education rather than in the context of medical or health professions education. Ball [5] refined elements of PCK for mathematics education, Mishra and Koehler [6] extended the PCK framework to include technology knowledge with the Technology, Pedagogy, and Content Knowledge (TPACK) framework, and Fraser [7] considered the application of PCK for science lecturers in an undergraduate setting. Substantial funding has been invested in the development of PCK measurement tools and PCK professional development programs for early educators [8]. Ironically, although the originator of PCK, Lee Shulman, spent a large portion of his career leading up to the introduction of PCK in part as a faculty member at Michigan State University’s medical school, the medical and health professions education community has not played an integral role in the development or integration of PCK.

## PCK Conceptualization

Shulman formally introduced PCK as the specialized expertise of teachers to transform subject matter content into comprehensible forms for learners [9]. Shulman described PCK as the often-tacit ability of an educator to know what to teach, how to teach, how to engage students, and how to deal with students’ learning difficulties [9, p. 8]. PCK recognizes that the unique competencies of skilled teachers encompass subject matter understanding coupled with pedagogical skills and an awareness of their students’ needs, which collectively enable teachers to design and implement effective instructional strategies [10]. An educator might exhibit PCK in the following process: (1) identify that a student has a misconception or knowledge gap that is either pre-existing or the result of ineffective instruction, (2) determine what the misconception or knowledge gap is, and (3) decide on and implement a pedagogical approach for addressing the misconception or closing the knowledge gap.

## Learning Context and PCK

Absent from the original conceptualization of PCK but found in later modifications was the importance of context, such as curriculum objectives and assessment, the attributes of the location in which learning is taking place, and the role of teachers’ orientation toward the subject area, particularly science [11, 12]. The contexts of teaching in medical and health professions education are varied and extend beyond the typical science classroom envisioned in the early discussions of PCK. The lecture hall, problem-based learning session, and clinic are some of the many settings in medical and health professions education in which PCK may be manifested. Further exploration and discussion around the notion of PCK resulted in a Consensus Model [10] and then a Refined Consensus Model [13] a few years later, both of which provide a more nuanced conceptualization of PCK and emphasize the importance of the learning context.

## The Refined Consensus Model of PCK

**Fig. 1:**
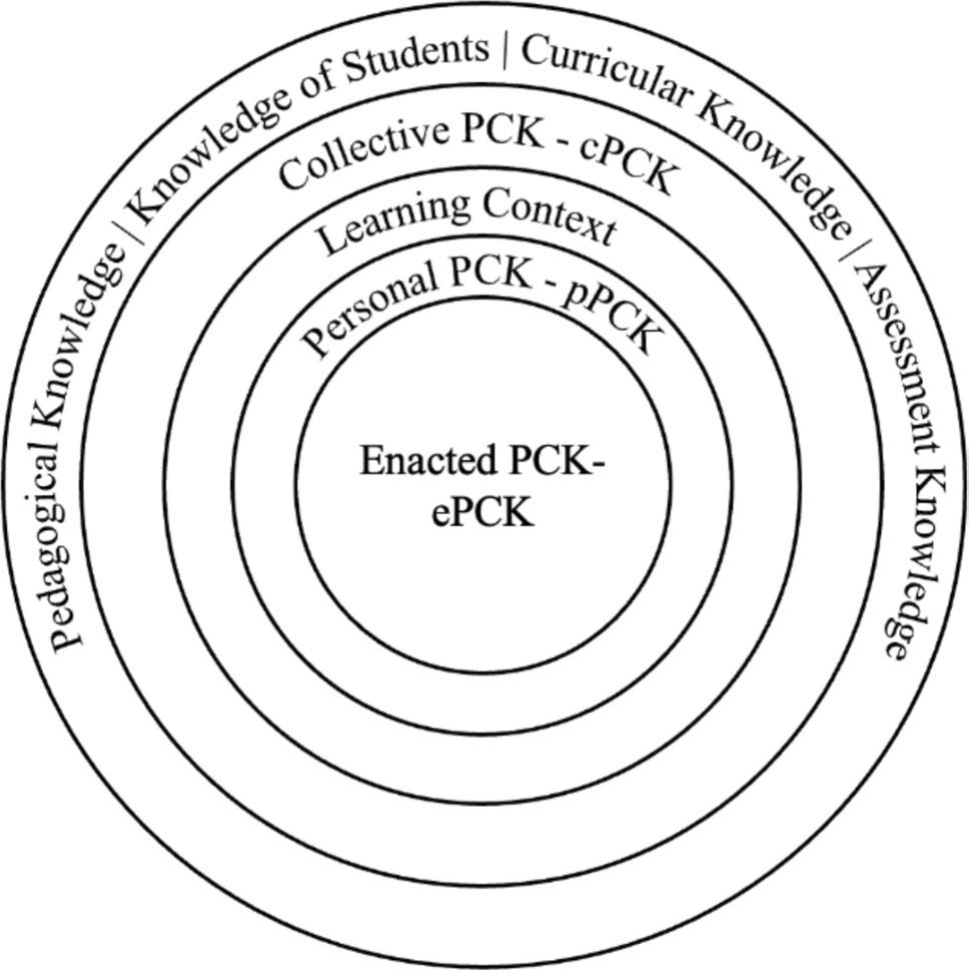
The Revised Consensus Model of PCK. Image sourced and modified from Carlson et al. [13]

The Refined Consensus Model of PCK breaks PCK into component parts, each of which informs the others (see Fig. 1). Enacted PCK (ePCK) is at the core of the model and represents the instructional decisions related to teaching explicit content to specific learners within a defined learning context. These decisions can be made during instruction or during planning and reflection. Personal PCK (pPCK) is a library of knowledge and skills from which an educator may draw to produce an ePCK. An individual’s pPCK can be built up through teaching experiences, input from colleagues, or professional development. There is a two-way exchange between ePCK and pPCK. In a moment of instruction, an educator may realize the need to present material in a different way, and, pulling from their pPCK library, modify their instruction to meet the needs of the learners. The success of this instance of ePCK is an experience that impacts the instructor’s pPCK. Wrapped around pPCK and ePCK is the learning context. The learning context is comprised of the attributes of the learning environment, learners, and educational climate that act as moderators of the educator’s instructional choices. Existing beyond the learning context is collective PCK (cPCK). This contains the knowledge and skills shared by a profession or group of professionals. This shared knowledge may exist in academic journals or in a single institution’s agreed upon curricular choices or community of practice. Thus, cPCK may include best practices for teaching using active learning or an agreed- upon manner of facilitating a problem-based learning session. Finally, the outermost circle contains the different professional knowledge bases. While these professional knowledge bases are essential in any application of PCK, they capture more generic knowledge. For example, content knowledge at this level is mastery of discipline-specific knowledge without any connection to pedagogy. Curricular knowledge includes the principles of sequencing lessons to build up to student mastery, but this knowledge is not content specific.

To summarize the elements of the Refined Consensus Model of PCK, ePCK is exhibited in a moment of decision, planning, or reflection in the complex interplay between an educator and learners. pPCK is the personal library of knowledge and skills that an educator pulls from when producing ePCK. The learning context informs the knowledge and skills pulled from pPCK to produce ePCK, and cPCK is the broader, shared knowledge and skills about teaching held by members of the profession or by a local group of professionals. The core elements of the professional knowledge base are the foundational blocks for instruction that, if expertly combined, may result in ePCK.

## Pivot and Preparation

Further nuance is added when we differentiate within ePCK based on timing [10]. An ePCK *pivot* is an in-the-moment decision of an instructor and affects teaching that occurs in real time. ePCK *preparation* occurs prior to a learning opportunity; an instructor considers common misconceptions or plans teaching strategies that preemptively address areas where a particular group of learners may encounter difficulties. Such preparation is an application of knowledge found in the instructor’s pPCK. An ePCK *pivot* can result in ePCK *preparation* if the instructor learns from an in-the-moment ePCK *pivo*t that learners have a common misconception or that a particular teaching strategy was ineffective and then adjusts how they teach in the future. If reflected upon, both pivots and preparation can become part of the pPCK library of the instructor.

In the example scenario we started with, the educator exhibited an ePCK pivot when an in-the-moment decision was made to reiterate content or use a think-pair-share activity. ePCK preparation occurs in the initial scenario if the instructor subsequently modifies the teaching approach, perhaps adding clarifying diagrams, building in an additional learning activity, or modifying the question so that the next time the session is presented, the learners will be more likely to demonstrate evidence of being able to apply the knowledge or skill. Reflection on the success of an ePCK pivot may result in the development of the instructor’s pPCK, essentially adding to the instructor’s teaching repertoire. The instructor’s pPCK then acts as a repository of pedagogical decisions that are drawn from to prepare for a future lesson, an act of ePCK preparation. These ePCK pivots and ePCK preparation can be seen across the diverse educational settings found in medical and health professions education.

## Example 1: Clinical Setting

Clinical experiences are used widely in medical and health professions education to develop learners’ clinical knowledge, skills, and behaviors in the context of a real-world healthcare setting [14]. Learners have opportunities to interact with patients and discuss cases with other learners, members of the medical team, and an attending physician.

Consider this scenario demonstrating ePCK: while in a clinic, a trainee has a misunderstanding that leads to an incorrect diagnosis of a patient. The attending physician recognizes the error as being rooted in the trainee’s misconception of a disease process. Because of the time limitations inherent in patient care and the need to tend to patient concerns, the attending physician must move quickly. The attending physician asks the trainee to contrast this case to a previous case observed that morning. The trainee then identifies differences between the two cases, and the attending explains how these differences lead to an alternative diagnosis. In this scenario, the attending has demonstrated an ePCK *pivot* by identifying an error (misdiagnosis), determining the knowledge that needed to be reinforced (disease process), and selecting a pedagogical strategy to close the knowledge gap (contrasting with prior case), all in the moment the error occurred (pivot) while considering what is feasible in the clinical setting.

Later, the attending physician reflects on this encounter and determines that a series of scaffolded questions could be used to close the knowledge gap just as effectively as the case comparison and would not require the trainee to have already seen certain types of patient cases. This would make scaffolded questioning a more reliable pedagogical choice. Doing so, the attending has exhibited ePCK *preparation* by planning scaffolded questions (a pedagogical decision) ahead of time (preparation) based on a known knowledge gap (identified student misconception or knowledge gap) that would enable future trainees to learn specific content while considering the differential nature of a given trainee’s experience working in a real clinical environment.

## Example 2: PBL Session

Problem-based learning (PBL) is a student-centered teaching method in which small groups of learners work together systematically to understand the science underlying a patient’s case, diagnose a patient presented in a medical case, or answer an authentic question. The approach gives students opportunities to develop self-directed learning habits while problem solving in a collaborative setting [15, 16]. Ideally, a PBL facilitator engages minimally with the PBL group of learners, who are responsible for managing the process and content of the session.

As an example of ePCK, consider this: during a PBL session occurring early in medical school, a facilitator notices that students are discussing answers to the questions they generated in a previous session. However, the discussion contains mainly simple, surface- level report-outs of answers. The facilitator notes that for PBL to work best, answering questions should be the starting point for more in-depth, student-driven discussions. The facilitator recognizes that the learners are not processing the case at the expected level of depth and may not know that their discussion should be improved. The facilitator also understands their role is to ensure that the PBL process is implemented with fidelity and not to contribute content knowledge to the discussion. The facilitator decides to pause the session between questions to encourage a deeper discussion of findings and invites the leader of the session to prompt this deeper discussion throughout. Additionally, PBL facilitators typically provide a reflective end-of-the-session prompt to which all learners respond. The facilitator in this case had planned to ask learners what they enjoyed most about today’s session. Instead, the facilitator recognizes the concluding prompt as an opportunity to have learners reflect on the depth of their discussion. At the end of the session, the facilitator says, “I noticed that in the beginning of the session the depth of content coverage was superficial. I would like you to share what you think the group should change going forward to improve the level of detail and depth of future discussions.” By responding to the prompt, learners were able to identify that they had workload challenges and were hesitant to contribute more to the discussion for fear of being perceived as monopolizing the time. As a result of their reflection, the learners had an open discussion about these challenges and encouraged one another to contribute more next time without fear of being perceived negatively. In this scenario, the facilitator observed an issue with the depth of content that was being discussed (identified a deficiency in how PBL was being implemented), potentially leaving gaps in what students were learning (content concern), and decided to intervene in a manner that fit within the defined role of a facilitator (contextual constraint) by prompting greater discussion depth and asking a related reflective prompt (pedagogical decision). This in-the-moment decision is an example of an ePCK *pivot* in the context of a PBL session.

Reflecting on this experience, the facilitator considers what other process errors may occur during a PBL session. The facilitator then develops a set of prompts that could be used to address these different PBL process errors. Doing so, the facilitator is now prepared to prompt learners to reflect on and improve their implementation of the PBL process. This prompt development to address future potential issues in PBL implementation is ePCK *preparation*.

## PCK in Medical and Health Professions Education

The relevance of PCK as a unique domain of educator competencies has not been taken up broadly as a framework to enhance medical educator effectiveness in classrooms and clinical learning settings. Even when the TPACK model has been applied to medical education, the focus has been on integrating technology with teaching and not the underlying concept of PCK [17]. This knowledge-to-practice gap may stem from the historical use of passive lecturing, which did not require educators to exhibit pedagogical knowledge, much less PCK, in the ways described. As education in these fields shifts toward more active engagement of learners, the need for faculty to develop PCK has grown. However, many educators are expected to “figure out” how to teach complex ideas and skills to learners in the absence of professional development related to general pedagogical knowledge and teaching practices. In nursing education, Crider [18] noted that novice nurse educators, including those in clinical settings, are expected to develop the competencies encompassed within PCK on their own, without the benefit of educator development. Medical and health professions educators typically have highly specialized content knowledge and skills but are unlikely to have had formal preparation in teaching strategies or curricular design [19, 20]. Without this formal training, instructors may be left to develop their instructional acumen on their own.

Adding explicit consideration of PCK into the context of medical and health professions teaching and learning, and educator professional development, extends and enriches descriptions of desirable educator competencies and attributes already outlined in the literature. As noted by Quirk and Harden, teachers’ toolkits should contain a variety of approaches (all of which have strengths and weaknesses) that can be deployed depending on the situation [21]. Harden and Laidlaw summarized key technical and “approaches to teaching” competencies and attributes that characterize effective teachers [1, p. 10]. They addressed many aspects of general pedagogical and PCK, including choosing appropriate teaching and facilitation methods, managing student learning in a variety of settings, and applying appropriate decision-making skills and best evidence-based approaches. The authors also noted that most faculty development programs focus mostly on technical teaching competencies, rather than on faculty members’ approaches to their own teaching and professionalism as educators. The importance of building medical teachers’ general pedagogical knowledge was further highlighted by McLeod et al., who reported positive outcomes from a pilot study to transform educators’ tacit into explicit pedagogical knowledge, but the authors did not explore educators’ content-area-specific and in-the-moment pedagogical moves that fall under the domain of PCK [22]. While aspects of PCK have been put forth in the medical and health professions literature, considering PCK in its entirety as a framework for understanding quality teaching provides a useful synthesis and extension of this prior work.

The PCK framework can be transformative in serving as a basis for continuing educator professional development or for training future faculty interested in a career in academia. PCK succinctly defines the tacit knowledge of an educator to educate well, providing a framework for designing educator professional development opportunities, expert-educator mentoring programs, and the creation of resources to enhance PCK development. Lamb and Firestone [23] suggested that partnering medical school educators with science educators, who would contribute general pedagogical knowledge and PCK, might enhance outcomes for a broader range of learners in medical education. They proposed the development of medical education–specific PCK through such partnerships and shared knowledge. Additionally, the collaborative development of detailed case descriptions in which PCK is exhibited has been proposed as a method for capturing and demonstrating PCK in specific learning scenarios [24, 25]. These approaches can be useful for developing pPCK and contribute to the broader development of cPCK. To facilitate medical and health professions educators in the development of their pPCK, we have also created a brief reflection tool that guides an educator through the steps of ePCK.

## ePCK Tool for pPCK Development

Prior work has sought to capture ePCK for the purpose of portraying it in a way that others can develop their own pPCK [24, 25]. Consistently, these tools require educators to reflect on their teaching practice. Reflection is an important process when improving one’s teaching practice [26]; therefore, with the same intent as prior work in PCK development, we provide a tool that can be used reflectively by medical and health professions educators interested in enhancing their own PCK. The tool contains a series of prompts that can be used either reflectively or prospectively to better understand and potentially develop an educator’s pPCK to then inform their ePCK.

Table 1 displays this tool completed for the three venues used in this paper (lecture hall, clinic, PBL session). The user is walked through the fundamental process of exhibiting ePCK: (1) recognizing the learning context and its potential constraints and affordances, (2) identifying that a student has a misconception or knowledge gap that is either pre-existing or the result of ineffective instruction, (3) determining specifically what the misconception or knowledge gap is, and (4) making a pedagogical decision. If the decision is in the moment of instruction, then it corresponds to a *pivot*; if the decision is made prior to the learning experience or in reflection on a learning experience, then it is *preparation*.

**Table 1:** ePCK tool examples

Topic	Learning context	Gap/misconception identification	Content identification	Pedagogical move	
High-level; body part/topic	Clinic/classroom/special session type (e.g., PBL), description of learners (level); if in clinic, consider adding information about the patient.	What triggered you to recognize a gap or misconception existed?	What content gap/misconception did the learner’s response reveal?	Pivot—What did you do to address the gap/misconception in the moment?	Preparation—How did you plan to modify your instruction/presentation to address the gap/misconception in the future?
Didactic session topic	First year medical students; lecture hall with stadium seating. Limited timeframe to cover specific learning objectives.	Learners were given a MCQ with three options, and 50% of the learners answered incorrectly.	Learners did not have sufficient prior knowledge to understand some of the session content, and therefore many learners did not select the correct MCQ answer.	I reiterated and expanded on the information from prior slides to explain why options A and B are incorrect and why C is the best answer. There was not enough time to do much else due to the time constraints of the session.	I revised the content of a prior slide in which treatment options are described; added a table that learners would complete to match treatment options to conditions.
Clinical teaching (moderate risk chest pain)	Clinic or other outpatient office visit (can also be inpatient or emergency department)	I observed the trainee misdiagnosing chest pain, with moderate risk of acute coronary syndrome as simply gastroesophageal reflux disease.	The trainee had a misconception in their thinking about disease process; specifically, they did not recognize that patients with chest pain must be risk stratified and that the presence of gastroesophageal reflex disease does not exclude cardiac disease.	Once I identified the misconception, I asked the trainee to contrast this case to a previous case observed while rounding. The trainee then identified differences between the two cases and I explained how these differences lead to a higher risk of acute coronary disease that must be evaluated.	I thought about some scaffolding questions that would not rely on the trainee having a comparator case previously on rounds.
PBL process	PBL session; 10 first -year medical students; PBL day 2, in which learners present answers to questions developed on PBL day 1; urban medical school; small group room.	Facilitator observes low Blooms’ level discussion, with students purely answering original questions. Students do not recognize that they are not meeting expectations for the quality of their discussions.	As students are trained to develop learning questions when they first receive a case, they can easily develop habits based on believing that answering the initial questions is sufficient.	In the session, I encouraged the leader to probe more because the depth was not sufficient. For the end-of-session prompt, I said, “I noticed in the beginning of the session that the depth of content coverage was not sufficient. I would like you to share what was causing this and/or what you think the group should change going forward.”	I developed additional prompts specifically related to PBL process issues that can be used in the future.

The ePCK tool is meant to be completed by an educator either after they have completed an ePCK pivot or during planning to capture ePCK preparation. By reflectively capturing these instances of ePCK, the educator completing the tool can solidify in their own mind why they made the educational decision they did, building up their own pPCK. A completed ePCK tool is useful for others to review as well. Doing so, educators can learn from the experiences of others, potentially contributing to their pPCK and the cPCK at their institution or beyond.

## Conclusion

The concept of PCK has existed for decades. Although its roots are found in Shulman’s time at Michigan State University’s medical school, PCK has received little attention in the medical and health professions education community. This absence may exist because of the historical focus on content delivery during lecture rather than active engagement for learning. As institutions emphasize active learning, the need for faculty development of PCK, and therefore consideration of what PCK is, has grown. To optimize learning, educators must notice instructional mismatches between an approach being used and its effectiveness with learners, including identifying misconceptions or barriers to learning, identifying the source of learning gaps or instructional ineffectiveness, and flexibly adjusting the pedagogical approach they take. In short, medical educators must possess and exhibit PCK. Like any skill, PCK can be developed with conscientious effort and reflection. Therefore, we have presented PCK in a medical and health professions education context and provided a reflective tool useful for capturing instances of ePCK with the intent of bridging this knowledge-to-practice gap.

In addition to individual development, PCK as a framework has broader implications. The cPCK element of PCK is the shared understanding of how to educate at a broader level, such as an entire institution. By developing individuals’ ePCK and pPCK, the cPCK can gradually shift. By viewing teaching as something to be developed through a PCK lens, institutions can design targeted continuing educator professional development opportunities to support individual growth as an educator. Incentivizing high-quality teaching, as exhibited by an individual exercising ePCK, would encourage faculty to take the extra step of reflecting on their practices. These incentives can take numerous forms, including awards or treating teaching as a unique pathway for promotion. By recognizing PCK as a framework for understanding the ability of an educator to educate well and designing opportunities and incentives for PCK development, it is possible that medical and health professions education can move forward effectively and efficiently for the benefit of our learners and those they will care for.
